# MnO_2_ prepared by hydrothermal method and electrochemical performance as anode for lithium-ion battery

**DOI:** 10.1186/1556-276X-9-290

**Published:** 2014-06-10

**Authors:** Lili Feng, Zhewen Xuan, Hongbo Zhao, Yang Bai, Junming Guo, Chang-wei Su, Xiaokai Chen

**Affiliations:** 1 Engineering Research Center of Biopolymer Functional Materials of Yunnan, Yunnan Minzu University, Kunming 650500, China; 2 Key Laboratory of Chemistry in Ethnic Medicinal Resources, State Ethnic Affairs Commission & Ministry of Education, Yunnan Minzu University, Kunming 650500, China; 3 College of Civil Engineering, Kunming University of Science & Technology, Kunming 650500, China

**Keywords:** Lithium-ion battery, Manganese dioxide, Hydrothermal method, Anode materials

## Abstract

Two α-MnO_2_ crystals with caddice-clew-like and urchin-like morphologies are prepared by the hydrothermal method, and their structure and electrochemical performance are characterized by scanning electron microscope (SEM), X-ray diffraction (XRD), galvanostatic cell cycling, cyclic voltammetry, and electrochemical impedance spectroscopy (EIS). The morphology of the MnO_2_ prepared under acidic condition is urchin-like, while the one prepared under neutral condition is caddice-clew-like. The identical crystalline phase of MnO_2_ crystals is essential to evaluate the relationship between electrochemical performances and morphologies for lithium-ion battery application. In this study, urchin-like α-MnO_2_ crystals with compact structure have better electrochemical performance due to the higher specific capacity and lower impedance. We find that the relationship between electrochemical performance and morphology is different when MnO_2_ material used as electrochemical supercapacitor or as anode of lithium-ion battery. For lithium-ion battery application, urchin-like MnO_2_ material has better electrochemical performance.

## Background

Manganese dioxides with diverse crystal morphologies are attracting a lot of attention because of their physical and chemical properties and wide applications in catalysis [1], biosensors [2], water treatment [3,4], electrochemical supercapacitors [5-9], and so on. Up to now, various MnO_2_ crystals with different morphologies such as nanosphere [10,11], nanorod [12,13], nanowire [13], nanoflower [13,14], nanotube [15], pillow-shape [4], urchin-like [10,16], hollow nanosphere, hollow nanocube [3], and hollow cone [17] have been synthesized. MnO_2_ crystals were already used in water treatment, gas sensors, electrochemical supercapacitors, and so on. For example, hollow spherical and cubic MnO_2_ nanostructures prepared by Kirkendall effect showed good ability to remove organic pollutants in waste water [3]. Cao et al. had prepared pillow-shaped MnO_2_ crystals which could remove about 85% of the Cd^2+^ in waste water [4]. Zhang et al. had prepared MnO_2_ hollow nanospheres and nanowires used for ammonia gas sensor [2]. MnO_2_ hollow nanospheres were found to exhibit enhanced sensing performance to ammonia gas at room temperature compared with MnO_2_ nanowires. Ma et al. had prepared urchin-shaped MnO_2_ and clew-like-shaped MnO_2_ used for electrochemical supercapacitors [6]. They found the electrochemical performances differed with various morphologies, and clew-like MnO_2_ nanospheres had higher capacitance and lower charge-transfer resistance due to their incompact structure. However, the application researches of MnO_2_ as anode for lithium-ion battery were relatively few.

MnO_2_ nanomaterials are recognized as anode materials since three-dimensional (3d) transition metal oxides (MO, where M is Fe, Co, Ni, and Cu) were proposed to serve as high theoretic capacity anodes for lithium-ion battery by Poizot et al. [18]. Before that, MnO_2_ nanomaterials were usually used to prepare LiMn_2_O_4_ crystals as cathode for lithium-ion battery [19,20]. Chen's research group has made great contributions on the research of anode for lithium-ion battery [21,22]. Nevertheless, compared to the intensive investigation on Fe_2_O_3_, Fe_3_O_4_, SnO_2_, CoO, and so on [23-28], the application investigation of MnO_2_ nanomaterials on anodes for lithium-ion battery is still immature, although the investigations on their preparation are plentiful.

The research on MnO_2_ anode is relatively complex because MnO_2_ exists in several crystallographic forms such as α-, β-, γ-, and δ-type. For example, Zhao et al. [22] reported γ-MnO_2_ crystals with hollow interior had high discharge capacity as 602.1 mAh g^−1^ after 20 cycles. Li et al. [15] found α-MnO_2_ with nanotube morphology exhibited high reversible capacity of 512 mAh g^−1^ at a high current density of 800 mA g^−1^ after 300 cycles. Thus, from the above two examples, we could summarize that the electrochemical performance of MnO_2_ crystals has relationship both with the crystallographic forms and with the morphologies. Therefore, the researches on the relationship of electrochemical performance with the morphologies and the relationship of electrochemical performance with the crystallographic forms are very essential.

In the present work, to enrich the relationship between electrochemical performances and morphologies, two α-MnO_2_ crystals with caddice-clew-like and urchin-like morphologies were prepared by hydrothermal method. For lithium-ion battery application, urchin-like α-MnO_2_ crystal with compact structure was found to have better electrochemical performance.

## Methods

### Synthesis and characterization of MnO_2_ micromaterials prepared by hydrothermal method

All reagents purchased from the Shanghai Chemical Company (Shanghai, China) were of analytical grade and used without further purification. The MnO_2_ micromaterials were prepared using the similar method described by Yu et al. [6] with some modifications. To prepare caddice-clew-like MnO_2_ micromaterial, 1.70 g MnSO_4_ · H_2_O was dissolved in 15-mL distilled water with vigorous stirring. When the solution was clear, 20-mL aqueous solution containing 2.72 g K_2_S_2_O_8_ was added to the above solution under continuous stirring. Then, the resulting transparent solution was transferred into a Teflon-lined stainless steel autoclave (50 mL) of 80% capacity of the total volume. The autoclave was sealed and maintained at 110°C for 6 h. After the reaction was completed, the autoclave was allowed to cool to room temperature naturally. The solid black precipitate was filtered, washed several times with distilled water to remove impurities, and then dried at 80°C in air for 3 h. The obtained caddice-clew-like MnO_2_ micromaterial was collected for the following characterization.

Urchin-like MnO_2_ micromaterial was prepared by the similar method, while after adding 1.70 g MnSO_4_ · H_2_O and 2.72 g K_2_S_2_O_8_ into 35-mL distilled water, 2 mL H_2_SO_4_ was then added. Subsequently, the solution was transferred into a Teflon-lined stainless steel autoclave (50 mL), and the autoclave was sealed and maintained at 110°C for 6 h as well. After the reaction was completed, the autoclave was allowed to cool to room temperature naturally. The solid black precipitate was filtered, washed several times with distilled water to remove impurities, and then dried at 80°C in air for 3 h.

The crystallographic structures of the products were determined with X-ray diffraction (XRD) which were recorded on a Rigaku D/max-2200/PC (Rigaku, Beijing, China) with Cu target at a scanning rate of 7°/min with 2*θ* ranging from 10° to 70°. The morphological investigations of scanning electron microscope (SEM) images were taken on a field emission scanning electron microscope (FESEM; Zeiss Ultra, Oberkochen, Germany).

### Electrochemical studies of MnO_2_ micromaterials

Electrochemical performances of the samples were measured using CR2025 coin-type cells assembled in a dry argon-filled glove box. To fabricate the working electrode, a slurry consisting of 60 wt.% active materials, 10 wt.% acetylene black, and 30 wt.% polyvinylidene fluoride (PVDF) dissolved in *N*-methyl pyrrolidinone was casted on a copper foil and dried at 80°C under vacuum for 5 h. Lithium sheet was served as counter and reference electrode, while a Celgard 2320 membrane (Shenzhen, China) was employed as a separator. The electrolyte was a solution of 1 M LiPF_6_ in ethylene carbonate (EC)-1,2-dimethyl carbonate (DMC) (1:1 in volume). Galvanostatical charge-discharge experiments were performed by Land electric test system CT2001A (Wuhan LAND Electronics Co., Ltd., Wuhan, China) at a current density of 0.2 C between 0.01 and 3.60 V (versus Li/Li^+^). Cyclic voltammogram (CV) tests were carried out on an electrochemical workstation (CHI604D, Chenhua, Shanghai, China) from 0.01 to 3.60 V (versus Li/Li^+^). Electrochemical impedance spectroscopy (EIS) measurements were performed on an electrochemical workstation (CHI604D, Chenhua, Shanghai, China), and the frequency ranged from 0.1 Hz to 100 kHz with an applied alternating current (AC) signal amplitude of 5 mV.

## Results and discussion

### Structure and morphology

**Figure 1:**
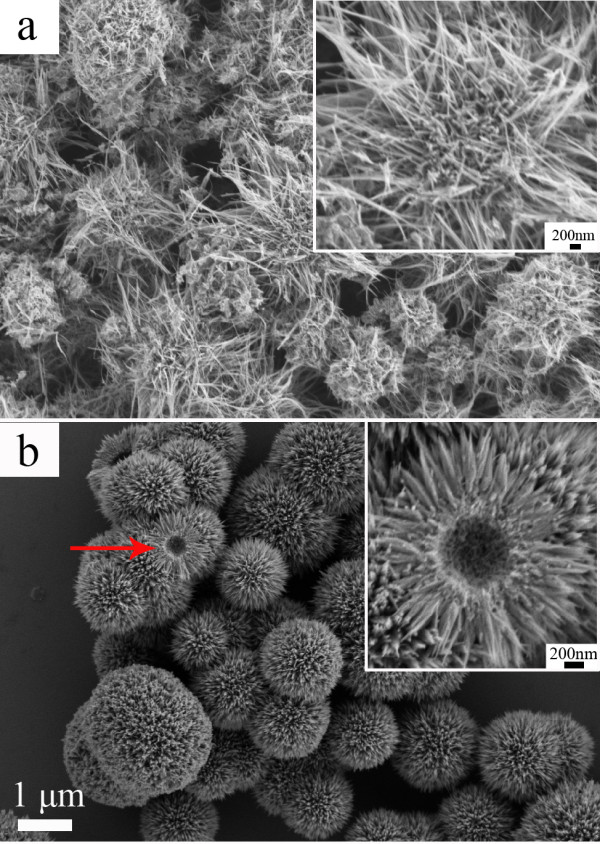
**SEM images of MnO**_**2 **_**samples obtained under (a) neutral and (b) acidic conditions.** The scale bar is 1 μm. The inset shows the enlarged SEM image of MnO_2_ sample and the scale bar is 200 nm.

**Figure 2:**
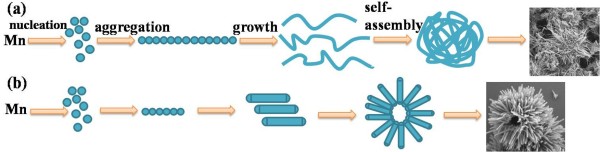
**The formation procedure of the MnO**_**2 **_**micromaterials. (a)** Caddice-clew-like and **(b)** urchin-like MnO_2_ samples.

The SEM images of the MnO_2_ micromaterials are displayed in Figure 1. The SEM study in Figure 1a indicates that the MnO_2_ prepared under the neutral reaction conditions is a nanowire 55 to 83 nm in diameter and several micrometers in length for average. Moreover, these nanowires aggregate into spherical shape with diameter of about 2 to 4 μm, and the MnO_2_ micromaterials are like a caddice clew. To mention the sample easily, we call this MnO_2_ micromaterial as caddice-clew-like MnO_2_. As shown in Figure 1b, when sulfuric acid was added as morphology modulation agent, the MnO_2_ micromaterial has a uniform sea-urchin-like shape with diameter of approximately 3 μm, which consists of several straight and radially grown nanorods with uniform length of about 1 μm. As indicated by the arrow in Figure 1b, the urchin-like MnO_2_ microsphere has a hollow interior. Figure 2 illustrates the possible formation processes for the MnO_2_ micromaterials. During the preparation of the MnO_2_ micromaterials, the K_2_S_2_O_8_ plays the role to oxidate the Mn^2+^ ion to MnO_2_. Firstly, the tiny crystalline nuclei of MnO_2_ are generated from Mn^2+^ by the oxidation in the supersaturated solution and grow into nanoparticles. The nucleation process could be regarded as

(1) $$\begin{array}{ll} {\mathrm{MnSO}}_{4} & +K_{2}S_{2}O_{8}+2H_{2}O\;\to {\mathrm{MnO}}_{2}+K_{2}{\mathrm{SO}}_{4}\\ & +2H_{2}{\mathrm{SO}}_{4}\;\mathrm{Reaction} \end{array}$$

As can be seen in Reaction (1), the reaction rate is pH dependent. Therefore, sulfuric acid is added to decrease the reaction rate, and the morphology can be modulated. When no sulfuric acid is used, these primary nanoparticles form quickly (shown in Figure 2(a)). Then, the tiny nanoparticles spontaneously aggregate into long nanowires. With minimizing interfacial energies, the nanowires wrap with each other incompactly to form caddice-clew-shaped MnO_2_ micromaterials. When sulfuric acid is added as morphology modulation agent, the nucleation process in Reaction (1) is suppressed. In this situation, it is not easy to form nanowires. Alternatively, short nanorods are formed (shown in Figure 2(b)). With minimizing interfacial energies, the nanorods self-assemble compactly to urchin morphology with a hollow interior. Thus, urchin-like MnO_2_ micromaterials are prepared. Therefore, sulfuric acid plays a crucial role in the morphology evolution due to its control of the nucleation rate of MnO_2_.

**Figure 3:**
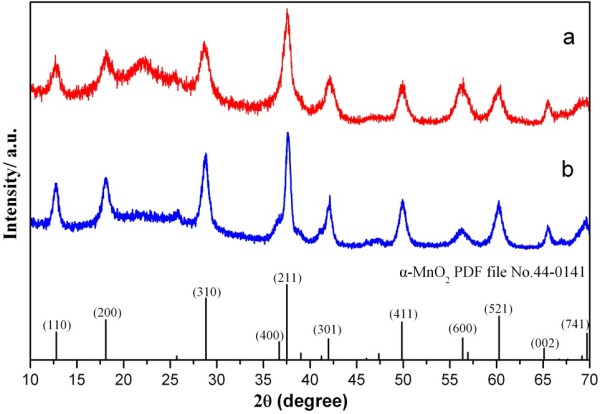
**The XRD patterns of MnO**_**2 **_**materials. (a)** Caddice-clew-like and **(b)** urchin-like MnO_2_ samples.

The XRD patterns of the MnO_2_ micromaterials are shown in Figure 3. As shown, the two samples had similar crystallographic structure. The diffraction peaks which appeared at 2*θ* = 12.7°, 18.1°, 28.8°, 37.5°, 42.1°, 49.9°, 56.2°, and 60.3° matched well with the diffraction peaks of (110),(200),(310),(211),(301),(411),(600), and (521) crystal planes of α-MnO_2_ standard data (JCPDS card PDF file no. 44-0141). Therefore, the two MnO_2_ micromaterials prepared by hydrothermal method were both α-MnO_2_, which was essential to evaluate the relationship between electrochemical performances and morphologies of MnO_2_ crystals as anodes for lithium-ion battery. As calculated, the lattice parameters of caddice-clew-like MnO_2_ are *a* = 9.7875 and *c* = 2.8600, which are highly identical to the standard values (JCPDS card PDF file no. 44-0141, *a* = 9.7847, *c* = 2.863). The cell volume of caddice-clew-like MnO_2_ is 273.97 Å^3^ which is also highly identical to the standard values (274.1 Å^3^),while the lattice parameters of urchin-like MnO_2_ are *a* = 9.8084 and *c* = 2.8483. According to the standard values, the crystal cell expands in a and b directions and contracts in c direction. The cell volume of urchin-like MnO_2_ is 274.02 Å^3^. The average size of the caddice-clew-like MnO_2_ crystal grains is calculated to be 32 nm according to the Scherrer equation *D* = *Kλ*/*β*cos*θ* using the strongest diffraction peak of (211) [*D* is crystal grain size (nm), *K* is the Scherrer constant (0.89), *λ* is the X-ray wavelength (0.154056 nm) for Cu Kα, *β* is the full width at half maximum (FWHM) of the peak (211), and *θ* is the angle of diffraction peak],while the measured diameter of caddice-clew-like MnO_2_ is 53 nm. The average size of the urchin-like MnO_2_ crystal grains is calculated to be 51 nm according to the Scherrer equation. The measured diameter of the short nanorods on urchin-like MnO_2_ is about 50 nm. As can be seen, the calculated crystallite size value of caddice-clew-like MnO_2_ crystal is a little smaller than the measured value, but the calculated crystallite size value of urchin-like MnO_2_ crystal is identical. Although the MnO_2_ micromaterials are in micrometer scale, they are confirmed to assemble by nanomaterials. Consequently, although the two MnO_2_ micromaterials are with identical crystal structure, they may have some difference in the electrochemical performance as the urchin-like MnO_2_ has the expanded lattice parameters.

### Electrochemical performance

**Figure 4:**
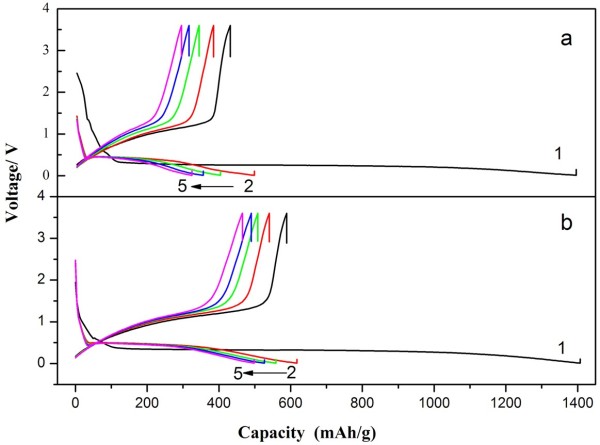
**Charge-discharge specific capacity-voltage curves of MnO**_**2 **_**anode materials in the potential range of 0.01 ~ 3.60 V at 0.2 C. (a)** Caddice-clew-like and **(b)** urchin-like MnO_2_ samples.

Figure 4 presents the typical charge-discharge voltage curves of the anodes (compared to the full battery) constructed from MnO_2_ micromaterials at 0.2 C rate in the voltage range of 0.01 to 3.60 V (vs. Li/Li^+^). For clarity, only selected cycles are shown. As shown, the two α-MnO_2_ micromaterials both have high initial discharge specific capacity as approximately 1,400 mAh g^−1^, while the theoretical discharge specific capacity is 1,232 mAh g^−1^. The extra discharge specific capacities of the as-prepared MnO_2_ micromaterials may result from the formation of solid electrolyte interface (SEI) layer which is known as a gel-like layer, containing ethylene oxide-based oligomers, LiF, Li_2_CO_3_, and lithium alkyl carbonate (ROCO_2_Li), during the first discharging process [29]. The discharge specific capacities of the as-prepared MnO_2_ micromaterials in the second cycle are 500 mAh g^−1^(caddice-clew-like MnO_2_) and 600 mAh g^−1^ (urchin-like MnO_2_), respectively. There is an attenuation compared to the initial discharge capacity. After the fifth cycling, the discharge specific capacities of the as-prepared MnO_2_ micromaterials are 356 mAh g^−1^ (caddice-clew-like MnO_2_) and 465 mAh g^−1^ (urchin-like MnO_2_), respectively. In the repeated charge-discharge cycling, the discharge specific capacity eventually decays to about 250 mAh g^−1^. Here, the large capacity loss may come from two facts: one is the capacity loss from the incomplete decomposition of SEI film, which happens in all 3d transition metal oxides including CuO, NiO, and Co_3_O_4_[29]; the other one is capacity loss caused by the electrode pulverization and loss of inter-particle contact or the particle with copper foil collector due to large volume expansion/contraction during repeated charging-discharging processes and severe particle aggregation, which is common in all transition metal oxides [30]. In fact, both the MnO_2_ micromaterials suffer from poor cycling stability of the discharge specific capacity. As usual, one effective way to mitigate the problem is to fabricate a hollow structure, as a hollow interior could provide extra free space for alleviating the structural strain and accommodating the large volume variation associated with repeated Li^+^ ion insertion/extraction processes, giving rise to improved cycling stability. However, the urchin-like MnO_2_ in this research indeed has a hollow interior but poor cycling stability. So, another effective strategy to improve the cycling stability is the need for the as-prepared MnO_2_ samples. For example, shell coating such as carbon coating, polypyrrole coating, and polyaniline coating is widely used to improve the cycling stability. Wan et al. prepared Fe_3_O_4_/porous carbon-multiwalled carbon nanotubes composite to promote cycle performance. Their excellent electrical properties can be attributed to the porous carbon framework structure, which provided space for the change in Fe_3_O_4_ volume during cycling and shortens the lithium ion diffusion distance [31]. Therefore, we are preparing polypyrrole coating MnO_2_ micromaterials to enhance the cycling stability.

In addition, a discharge plateau with wide and flat shape appears in all the discharge voltage curves. Urchin-like MnO_2_ micromaterial has a plateau at about 0.32 V from 120 to 1,100 mAh g^−1^ during the first discharging process and has a plateau from 50 to 360 mAh g^−1^ in the second cycling. The caddice-clew-like MnO_2_ micromaterial has similar discharge plateau. The discharge plateau may bring stable discharge current to the battery prepared by MnO_2_ micromaterials. According to the results of discharge specific capacity, urchin-like MnO_2_ micromaterial was better than caddice-clew-like MnO_2_ micromaterial.

**Figure 5:**
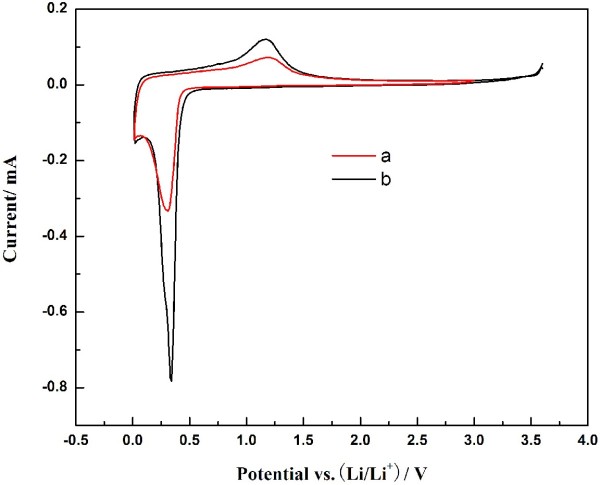
**Cyclic voltammograms of MnO**_**2 **_**materials.** After five charging-discharging cycles measured at a scan rate of 0.05 mV s^−1^in the potential range of 0.01 ~ 3.60 V. **(a)** Caddice-clew-like and **(b)** urchin-like MnO_2_ samples.

The cyclic voltammogram curves were tested to further investigate the electrochemical performances of the MnO_2_ micromaterials, as shown in Figure 5. In the CV curves, there is only a pair of redox peaks, indicating the one-step intercalation and deintercalation of lithium ion during the charging and discharging process. The reduction peak is at about 0.3 V, which corresponds well with flat discharge plateau in Figure 4. In this process, MnO_2_ is transformed to Mn, and Li^+^ is inserted into the anode to format Li_2_O. The reaction is as follows:

(2) $$\;{\mathrm{MnO}}_{2}+4\mathrm{Li}\to 2{\mathrm{Li}}_{2}O+\mathrm{Mn}\;\mathrm{Reaction}$$

The oxidation peak is at about 1.18 V, corresponding to the charging process of the lithium-ion battery. During this process, Mn can facilitate the decomposition of Li_2_O. The reaction of Li_2_O with Mn was as follows:

(3) $$2{\mathrm{Li}}_{2}O+\mathrm{Mn}\to {\mathrm{MnO}}_{2}+4\mathrm{Li}\;\mathrm{Reaction}$$

The current intensity of oxidation peak is much lower than that of reduction peak. The current intensity of reduction peak and oxidation peak for the urchin-like MnO_2_ material is 0.7828 and 0.1202 mA mg^−1^, respectively. The current intensity attenuation of oxidation peak indicates that Mn element could not completely convert to MnO_2_ during the charging process. The shapes of the CV curves for the MnO_2_ samples are similar, while urchin-like MnO_2_ material has higher peak intensity. The current intensity of reduction peak and oxidation peak for the caddice-clew-like MnO_2_ material is 0.3333 and 0.0712 mA mg^−1^, respectively. The asymmetry cyclic voltammogram curves in Figure 5 indicate that the discharging/charging process is irreversible.

To exclude the influence of the MnO_2_ micromaterial density on the electrode, we have normalized the CV curve in Figure 5. According to the results of galvanostatical charge-discharge experiments and CV tests, the urchin-like MnO_2_ micromaterial is more superior than caddice-clew-like MnO_2_ micromaterial. We presume the difference on electrochemical performance results from the morphology as both the MnO_2_ micromaterials have identical crystalline phase. Theoretically, nanomaterials with incompact structure are beneficial to improve the transmission rate and transfer ability of lithium ion. However, the discharge cycling stability of caddice-clew-like MnO_2_ micromaterial is poor. We guess the incompact structure may lead to easy electrode pulverization and loss of inter-particle contact during the repeated charging-discharging processes. A hollow structure which is another effective strategy to improve the cycling stability could provide extra free space for alleviating the structural strain and accommodating the large volume variation associated with repeated Li^+^ insertion/extraction processes. So, the relatively better discharge cycling stability may result from the hollow structure. In addition, the surface of urchin-like MnO_2_ is an arrangement of compact needle-like nanorods, which could improve the transmission rate and transfer ability of lithium ion. Therefore, the electrochemical performances of the MnO_2_ micromaterials indeed have relationship on their morphologies. The results suggest that the urchin-like MnO_2_ micromaterial is more promising for the anode of lithium-ion battery.

Compared to the literature [6], when MnO_2_ materials were used for electrochemical supercapacitors, caddice-clew-like MnO_2_ material had higher specific capacitance of 120 F g^−1^ and lower charge-transfer resistance, while the specific capacitance of urchin-like MnO_2_ material was about 48 F g^−1^. Moreover, they found the unique capacitance of caddice-clew-like MnO_2_ was mainly due to the incompact structure. Therefore, the relationship between electrochemical performance and morphology is different when MnO_2_ material is used as electrochemical supercapacitor or as anode of lithium-ion battery. For the application on lithium-ion battery, urchin-like MnO_2_ material is better.

**Figure 6:**
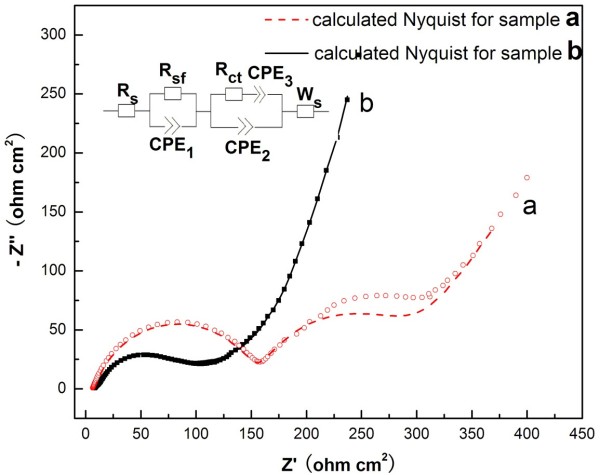
**Nyquist plot of Li/MnO**_**2 **_**cells after five charging and discharging cycles at open circuit voltage.** The frequency ranged from 0.1 Hz to 100 kHz with an applied AC signal amplitude of 5 mV. **(a)** Caddice-clew-like and **(b)** urchin-like MnO_2_ samples. Symbols represent experimental data and lines represent fitted spectra using equivalent circuit. The inset is the equivalent circuit.

In order to gain further understanding of the differences in the electrochemical performances, EIS testing was carried out. Figure 6 presents the EIS results for lithium cells after the fifth cycle at open circuit voltage. As shown in Figure 5(a), the impedance spectra of caddice-clew-like MnO_2_ consist of two oblate semicircles in high-to-medium frequency region and an inclined line in low-frequency region, while the two semicircles of urchin-like MnO_2_ are not easily distinguishable. The impedance spectra reflect several processes that take place in a series: Li migration through surface films, charge transfer, solid-state diffusion, and finally, accumulation of Li in the bulk of the active mass. An intercept at the *Z*_real_ axis in high-frequency region corresponds to the ohmic electrolyte resistance (*R*_s_). The first semicircle in the high frequency ascribes to the Li-ion migration resistance (*R*_sf_) through the SEI films. The second semicircle in the high-to-medium frequency ascribes to the charge transfer resistance (*R*_ct_). The inclined line at low-frequency region represents the Warburg impedance (*W*_s_), which is associated with lithium-ion diffusion in the active material [32,33].

The parameters of impedance spectra were simulated by ZSimpWin software, and the spectra had been fitted with an equivalent circuit shown in the inset of Figure 6. In the equivalent circuit of EIS, apart from the *R*_s_, *R*_sf_, *R*_ct_, and *W*_s_, the corresponding constant phase element (CPE) is used instead of pure capacitance due to the non-ideal nature of the electrode. The values of *R*_sf_ and *R*_ct_ calculated from the diameters of the high frequency and the high-to-medium frequency semicircles in the Nyquist plots for the electrodes are summarized in Table 1. The value of *R*_s_ for urchin-like MnO_2_ is 7.12Ω, while the value of *R*_s_ for caddice-clew-like MnO_2_ is 8.05Ω. Theoretically, the value of *R*_s_ for both Li/MnO_2_ cells should be the same as for the same electrolyte used here. The slight difference may be caused by the tiny difference in the battery package pressure by manual operation or the tiny difference in the amount of electrolyte added to the Li/MnO_2_ cells by manual operation. Considering the tiny difference in manual operation, the small difference of *R*_s_ is acceptable since the ohmic electrolyte resistances of the MnO_2_ micromaterials are similar. The *R*_sf_ and *R*_ct_ of the urchin-like MnO_2_ are much lower than that of the caddice-clew-like MnO_2_. It proves that the Li-ion migration resistance through the SEI films and charge transfer resistance of the urchin-like MnO_2_ are much lower than that of the caddice-clew-like MnO_2_. Here, the influence of the tiny difference in the battery package pressure and the amount of electrolyte on the *R*_sf_ and *R*_ct_ can be neglected. So, the urchin-like morphology is more favorable for lithium ion diffusion and transfer, and the reaction of MnO_2_ micromaterials with lithium ion is much easier.

**Table 1:** ***R***_**s**_**, *R***_**sf**_**, and *R***_**ct **_**calculated from Nyquist plots for the MnO**_**2 **_**materials**

	***R***_**s **_**(Ω cm**^**2**^**)**	***R***_**sf **_**(Ω cm**^**2**^**)**	***R***_**ct **_**(Ω cm**^**2**^**)**
a	8.05	121.40	146.90
b	7.12	94.66	43.64

a, caddice-clew-like MnO_2_ sample; b, urchin-like MnO_2_ sample.

## Conclusions

In summary, two MnO_2_ micromaterials with urchin-like and caddice-clew-like morphologies are prepared by hydrothermal method. Both the crystalline phases are α-MnO_2_, which is essential to evaluate the relationship between electrochemical performances and morphologies of MnO_2_ crystals as anodes for lithium-ion battery application. Both the as-prepared α-MnO_2_ exhibit high initial specific capacity, but the discharge cycling stability is poor. Just in case of this research, the urchin-like MnO_2_ material has better electrochemical performance. The results suggest that different morphologies indeed have influence on electrochemical performances of MnO_2_ micromaterials in the application of lithium-ion battery. This study also gives us advice to make shell coating on the as-prepared MnO_2_ micromaterials to improve the cycling stability.

## Competing interests

The authors declare that they have no competing interests.

## Authors’ contributions

The experiments and characterization presented in this work were carried out by LF, ZX, HZ, and YB. The experiments were designed by LF. The results of the experiments were discussed by LF, JG, CS, and XC. All authors read and approved the final manuscript.
